# How Does the First Molar Root Location Affect the Critical Stress Pattern in the Periodontium? A Finite Element Analysis

**DOI:** 10.30476/dentjods.2022.93271.1704

**Published:** 2023-06-01

**Authors:** Zahra Baghani, Reza Soheilifard, Sahar Bayat

**Affiliations:** 1 Dept. of Periodontics, Faculty of Dentistry, Sabzevar University of Medical Sciences, Sabzevar, Iran; 2 Dept. of Mechanical Engineering Hakim Sabzevari University Sabzevar, Iran; 3 Student, Dept. of Civil Engineering, Hakim Sabzevari University, Sabzevar, Iran

**Keywords:** Finite Element Analysis, Mechanical Stress, Molar, Periodontium, Tooth Root

## Abstract

**Statement of the Problem:**

The first molar root location plays a pivotal role in neutralization of forces applied to the teeth to prevent injury.

**Purpose:**

This study aimed to assess the effect of maxillary and mandibular first molar root location on biomechanical behavior of the periodontium under vertical and oblique loadings.

**Materials and Method:**

In this three-dimensional (3D) finite element analysis (FEA), the maxillary and mandibular first molars and their periodontium were modeled. The Young’s modulus and the Poisson’s ratio for the enamel, dentin, dental pulp, periodontal ligament (PDL), and cortical and cancellous bones were adopted from previous studies. The changes in maximum von Misses stress (MVMS) values of each component were analyzed.

**Results:**

The MVMS values were the highest in the enamel followed by dentin, cortical bone, cancellous bone, and PDL. The maxillary and mandibular first molars with different root locations and their periodontium showed different biomechanical behaviors under the applied loads.

**Conclusion:**

An interesting finding was that the stress concentration point in the path of load degeneration changed from the cervical third in dentin to the apical third in the cancellous bone, which can greatly help in detection of susceptible areas over time.

## Introduction

The periodontium plays a fundamental role in transfer of masticatory forces from the tooth to the alveolar bone, and force degradation [ 1 ]. From the clinical point of view, excessive occlusal forces can damage the periodontal tissue, temporomandibular joint, masticatory muscles, and pulp tissue [ 2 ]. However, from the biological point of view, animal studies have indicated that excessive loading is associated with the formation of pressure and tension sites in bone, depending on the magnitude and direction of the applied forces. The pressure sites are characterized by bone resorption, while the tension sites are characterized by bone remodeling [ 3 - 5 ]. The occurrence of the bone remodeling process depends on the maximum load and number of fixed daily cycles of load application [ 6 - 8 ]. It appears that the pattern and location of maximum stress concentration may differ depending on the root location of molar teeth. Therefore, pattern of stress distribution in teeth with different morphologies and biomechanical behavior of the components (which are not homogenous) should be studied in physiological occlusion and under maximum mastication force (MMF). Evidence shows that the teeth are more resistant to vertical loads. Thus, occlusal stress is considered as the main cause of dental injury [ 4 , 9 - 14 ]. It can traumatize the tooth at the stress concentration points and even lead to tooth fracture over time [ 15 - 16 ]. 

Detection of stress concentration points in the tooth structure and periodontium is not easy. A precise biomechanical model is required to analyze the causes of tooth fracture through a finite element analysis (FEA). FEA can reveal the qualitative responses to biomechanical stimuli [ 17 - 22 ]. It can simulate the clinical oral environment and stress-strain patterns [ 23 ], and is therefore, commonly used in dentistry for analysis of the teeth and the periodontium [ 24 - 29 ]. 

Borčić *et al*. [ 30 ] evaluated the three-dimensional (3D) pattern of stress distribution in a maxillary first premolar with two roots under two different loading conditions namely cusp to fossa occlusion, and cusp to fossa and cusp to marginal ridge occlusion, by applying oblique and vertical loads. They showed that vertical loads were dominant compared with tensile loads, and maximum concentration of vertical stress occurred at the dentinoenamel junction in the cervical third of all models. However, tensile stresses were recorded at the vestibular surface of the buccal cusps (about +3 MPa) and also in the central fossa of both models (about +28 MPa) in the cusp to fossa and cusp to marginal ridge occlusion. They only evaluated the stress distribution pattern in the tooth structure, and maximum stress concentration was noted in dentin and enamel in the cervical region [ 30 ]. It seems the location of roots can also affect these maximum stress points on the periodontium. Therefore, the pattern of stress distribution and degradation should be studied to find the actual points of stress accumulation and susceptible areas at which, the stress level might exceed the tolerance threshold of the tissue. Since first molars are the largest posterior teeth and have the most complex morphology [ 35 ], they play a fundamental role in distribution of masticatory forces [ 32 - 35 ]. 

Some recent studies [ 36 - 38 ] evaluated the pattern of stress distribution in the tooth structure, PDL, and interproximal contacts of restored and endodontically-treated maxillary and mandibular first molars by 3D FEA. Assessment of the biomechanical behavior of the teeth and stress distribution in the process of masticatory force degradation in single-rooted teeth and detection of points of maximum stress accumulation are a research priority [ 31 , 39 - 40 ]. Some other anatomical factors such as the number and location of roots in posterior multi-rooted teeth can also change the critical stress points. Thus, knowledge about the areas of maximum von Mises stress (MVMS) values can reveal areas susceptible to root fracture under parafunctional forces and indicate areas in need of precise occlusal adjustment. Therefore, knowledge about the areas susceptible to stress accumulation with respect to root location in posterior teeth is essential to minimize the risk of subsequent periodontal injury. Thus, this study aimed to assess the effect of number and location of roots of intact maxillary and mandibular first molars on biomechanical behavior of the teeth and periodontium under vertical (physiological and MMF) and oblique loadings. 

## Materials and Method

### Designing the 3D finite element model

A computed-tomography (CT) scan of molar teeth that met the inclusion criteria was used for this study. The inclusion criteria was defined as presence of an intact maxillary first molar with three normal roots and no restorative or endodontic treatment, presence of an intact mandibular first molar with two normal roots and no restorative or endodontic treatment, and normal morphology of the crown and root of mandibular and maxillary first molars according to the textbooks [ 41 ]. Teeth that did not meet the inclusion criteria were not used for modeling.

**Figure 1:**
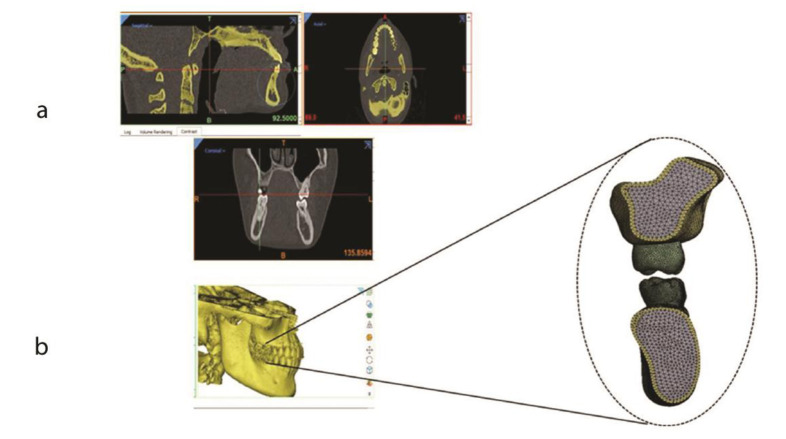
Segmentation process; **a:** Meshing of the mandibular first molar along with its attachment apparatus, **b:** Meshing of the maxillary first molar along with its attachment apparatus

Accordingly, the skull CT scan of a 31 year-old female with sound mandibular and maxillary first molars obtained by a high-resolution CT scanner (LOTUS *in vivo*, Iran) with the exposure settings of 45-90 kV, 0.18 mA, 80 × 200 mm field of view, 0.5 mm slice thickness, and 0.01 mm pixel size was used for this study. The CT data in DICOM format were imported to Mimics 19.0 software (Materialise, Leuven, Belgium mimics) to create 3D models of the enamel, dentin, dental pulp, PDL (0.25 mm), and cortical and cancellous bones (1 mm), and geometrically reconstruct the mandibular and maxillary first molar occlusal surfaces. The 3D model of the first molars with the surrounding bone was saved in STL format and imported to GEOMAGIC Studio 12.0 software to fix the errors of the model geometry, and was then imported to ANSYS19.1 software for meshing (Figure 1).

The designed model was then geometrically optimized, and the Young’s modulus and Poisson’s ratio for different components were defined. All components were considered homogenous, isotropic, and linearly elastic. The geometric models were then transferred to the FEA software for meshing. Trihedral parabolic solid elements were used for this purpose.

Table 1 presents the Young’s modulus and Poisson’s ratio for different components of the teeth and the attachment apparatus.

**Table 1:** Elastic properties of different components of the teeth and the attachment apparatus

Component	Young’s modulus (MPa)	Poisson’s ratio
Cortical bone [ 42 ]	15000	0.3
Dental pulp [ 43 ]	2	0.45
Dentin [ 44 ]	18600	0.31
Enamel [ 44 ]	84100	0.2
Lamina dura [ 42 ]	15000	0.3
Periodontal ligament [ 44 ]	70.3	0.45
Cancellous bone [ 42 ]	1500	0.3

The finite element model of the mandibular first molar comprised of 274,664 nodes and 169,503 elements while the finite element model of the maxillary first molar comprised of 243,714 nodes and 149,843 elements. To assess the pattern of stress distribution in the components based on the Von Mises stress criteria, the models were transferred to ANSYS 19.1 software (ANSYS Inc., Canonsburg, PA) (Figure 1B).

### Loading process

The loading process was adopted from the studies by Jiang *et al*. [ 37 ], Yoon *et al*. [ 45 ], and Yuan *et al*. [ 46 ], for vertical and lateral loadings (in order to be able to compare the results). According to Jiang *et al*. [ 37 ], vertical load was applied to 5 points in the occlusal surface of the maxillary first molar, and lateral forces were also applied to the lingual slope of the lingual cusp. Moreover, according to Yoon *et al*. [ 45 ], vertical load was applied to 5 points in the occlusal surface of the mandibular first molar that were in contact with the opposing tooth during mastication. According to Yuan *et al*. [ 46 ], lateral forces were applied to two points in the lingual slope of the buccal cusps at 45-degree angle relative to the longitudinal axis of the tooth. It should be noted that the magnitude of vertical and lateral loads was distributed among the loaded points. Therefore, three loading conditions were considered for FEA as follows:

### Loading condition 1

Axial load in an amount of 250 N [ 47 ] was applied to 5 points in the occlusal surface to simulate normal application of masticatory forces in the clinical setting. These points were the central fossa, mesial and distal marginal ridges, and the center of the buccal cusps in the mandibular first molar [ 45 ], and the central fossa, mesial and distal marginal ridges, and the center of the palatal cusps in the maxillary first molar [ 37 ].

### Loading condition 2

Oblique load in an amount of 100 N [ 11 ] with 45-degree angle was applied to simulate lateral forces applied during mastication in the clinical setting. The loading points were the internal slope of the buccal cusps of the mandibular first molar [ 46 ] and the internal slope of the palatal cusps of the maxillary first molar.

### Loading condition 3

**Figure 2:**
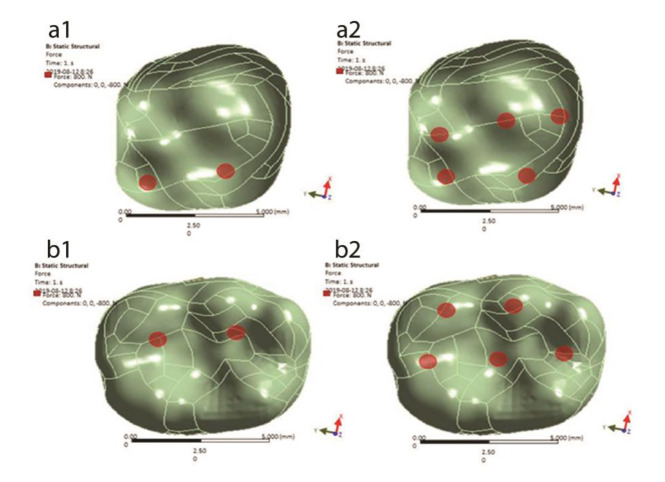
Loading points; **a1**: A force of 100 N was applied to the slope of the palatal cusps at 45-degree angle relative to the longitudinal axis of the maxillary first molar model, **a2:** A vertical force of 250 N was applied to 5 points in the maxillary first molar model, **b1:** A force of 100 N was applied to the slope of the buccal cusp at 45-degree angle relative to the longitudinal axis of the mandibular first molar model, **b2:** A vertical force of 250 N was applied to 5 points in the mandibular first molar model

Axial load in an amount of 800N [ 45 , 48 ] was applied to the same points mentioned above in the maxillary and mandibular first molars to simulate MMF (Figure 2).

Each load was separately applied to the mandibular and maxillary first molars. In total, the following six models were analyzed as Model 1: loading condition 1 on the mandibular first molar, Model 2: loading condition 2 on the mandibular first molar, Model 3: loading condition 3 on the mandibular first molar, Model 4: loading condition 1 on the maxillary first molar, Model 5: loading condition 2 on the maxillary first molar, and Model 6: loading condition 3 on the maxillary first molar. In all models, nodes in the bottom surface of the cortical bone were fully constrained.

## Results

**Figure 3:**
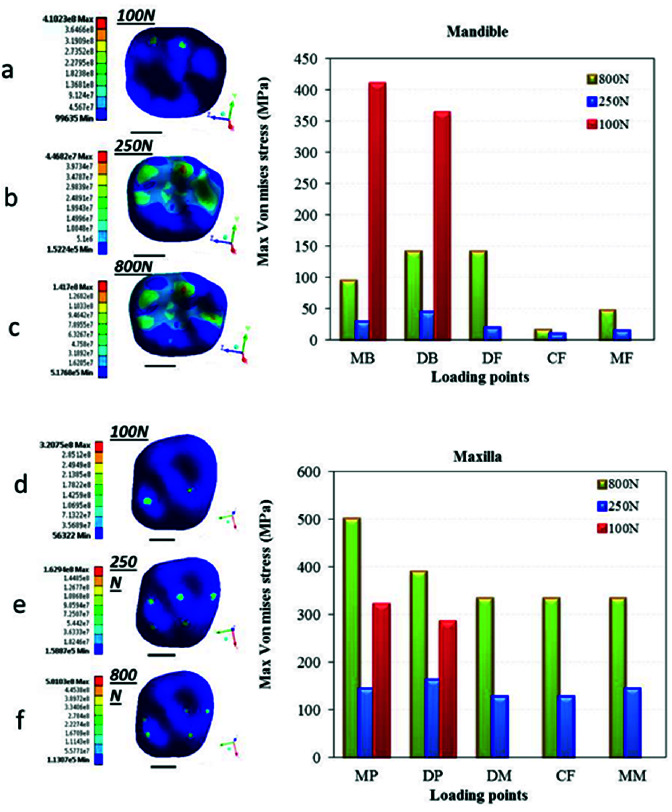
Von Mises stress distribution in the occlusal surface of maxillary and mandibular first molars in six models. Figures a, b and c present stress distribution in the occlusal surface of mandibular first molar under **a:** 100 N oblique load, **b:** 250 N vertical load and **c:** 800 N traumatic load. Figures d, e and f present stress distribution in the occlusal surface of maxillary first molar under d: 100 N oblique load, **e:** 250 N vertical load and **f:** 800 N traumatic load, MB: Mesiobuccal, DB: Distobuccal, DF: Distal fossa, CF: Central fossa, MF: Mesial fossa, MP: Mesiopalatal, DP: Distopalatal, DM: Distal marginal ridge, MM: Mesial marginal ridge

The MVMS values in the six models were analyzed in different tissues to find the effect of root location on susceptible high-stress points in the maxillary (with mesial, distal, and palatal root locations) and mandibular (with mesial and distal root locations) first molars and their periodontium. Figure 3 shows the results of loading of the occlusal surface, which is the first surface that receives the applied forces. Figures 3a, 3b, and 3c depict the pattern of stress distribution in the enamel of mandibular first molar under different loading conditions. Figures 3d, 3e, and 3f depict the pattern of stress distribution in the enamel of maxillary first molar under different loading conditions. To compare the results more accurately, the charts of MVMS values in the loading points were also drawn. As shown in Figure 3b, in vertical loading of the mandibular first molar, the maximum stress was concentrated in the distobuccal cusp (model 2); whereas, the maximum stress was concentrated in the mesiopalatal cusp in vertical loading of the maxillary first molar (model 5). The magnitude of this stress in the maxillary first molar was approximately 3 times the value in the mandibular first molar. 

In application of oblique loads, stress concentration in the mandibular first molar was mainly in the mesiob-uccal cusp (model 1); while, it was in the mesiopalatal cusp in the maxillary first molar (model 4). In addition, the magnitude of stress in the maxillary first molar was half the value in the mandibular first molar (Figures 3a and d). 

In application of MMF, no significant difference was noted regarding the stress concentration points, compared with the standard loading conditions. The only difference was that the magnitude of stress in both jaws was approximately 3 times the value in standard loading conditions (models 3 and 6). The magnitude of stress in the mandibular first molar under oblique loading was about 10 times the value in model 2 (standard vertical loading) and even higher than that in application of MMF (model 3). However, in the maxillary first molar, the maximum stress value was about 2 times higher than that in standard loading (model 5), and lower than that in application of MMF (Figure 3). 

**Figure 4:**
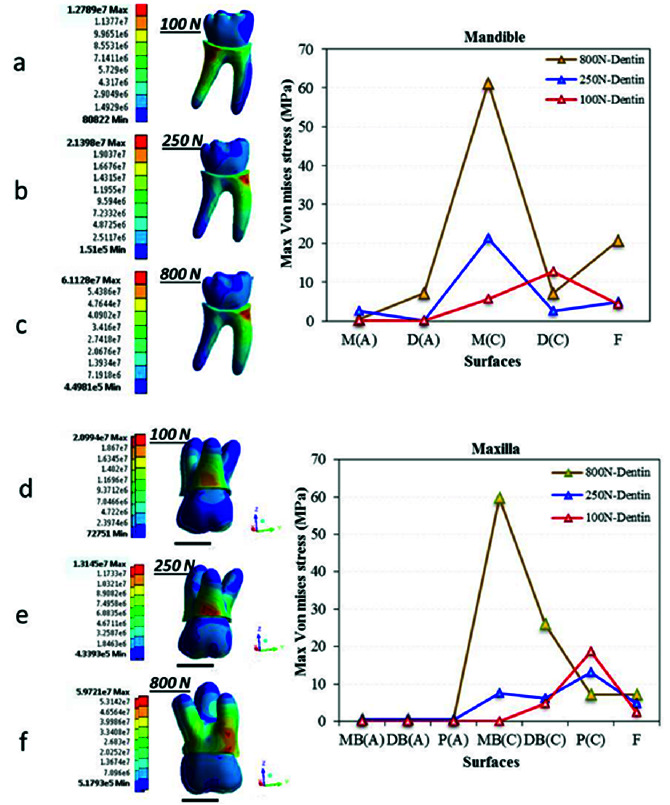
Von Mises stress distribution in dentin for the six models. Figures a, b and c present stress distribution in the mandibular first molar dentin under **a:** 100 N oblique load, **b:** 250 N vertical load and **c:** 800 N traumatic load. Figures d, e and f present stress distribution in the maxillary first molar dentin under **d:** 100 N oblique load, **e:** 250 N vertical load and **f:** 800 N traumatic load. Mandible: M(A): Mesial (Apex), D(B): Distal (Apex), M(C): Mesial (Cervical), D(C): Distal (Cervical), **F:** Furcation. (Maxilla: MB(A): Mesiobuccal (Apex), DB(A): Distobuccal (Apex), P(A): Palatal (Apex), MB(C): Mesiobuccal (Cervical), DB(C): Distobuccal (Cervical), P(C): Palatal (Cervical), F= Furcation)

Figure 4 shows the points of MVMS concentration in dentin, which is the second tissue that receives load after the enamel. In vertical loading of the mandibular first molar (models 2 and 3), loads were mainly distributed through the mesial root while in oblique loading (model 1), load transfer was mainly through the distal root. In the maxillary first molar (models 4 and 5), the maximum stress was noted in the palatal root, while in model 6 the maximum stress was found in the mesial root. The maximum stress in all models was at the cervical region, and its magnitude in oblique loading was higher than that in standard and MMF loading conditions, and was not significantly different between the maxillary and mandibular first molars (Figure 4). 

**Figure 5:**
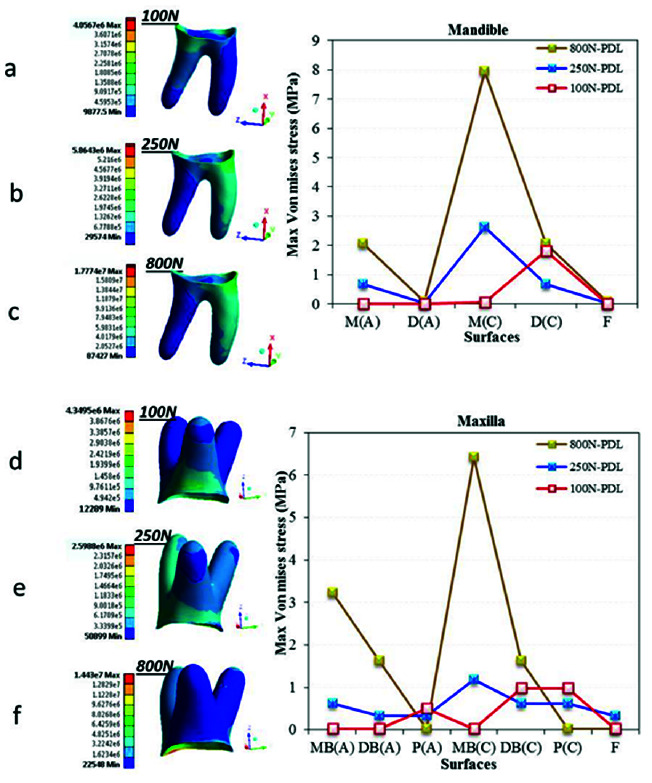
Von Mises stress distribution in the PDL for the six models. Figures a, b and c present stress distribution in the mandible under **a:** 100 N oblique load, **b:** 250 N vertical load and **c:** 800 N traumatic load. Figures d, e and f present stress distribution in the maxilla under **d:** 100 N oblique load, **e:** 250 N vertical load and **f:** 800 N traumatic load. (Mandible: M(A): Mesial (Apex), D(B): Distal (Apex), M(C): Mesial (Cervical), D(C): Distal (Cervical), F: Furcation). (Maxilla: MB(A): Mesiobuccal Apex, DB(A): Distobuccal (Apex), P(A): Palatal (Apex), MB(C): Mesiobuccal (Cervical), DB(C): Distobuccal (Cervical), P(C): Palatal (Cervical), F: Furcation)

**Figure 6:**
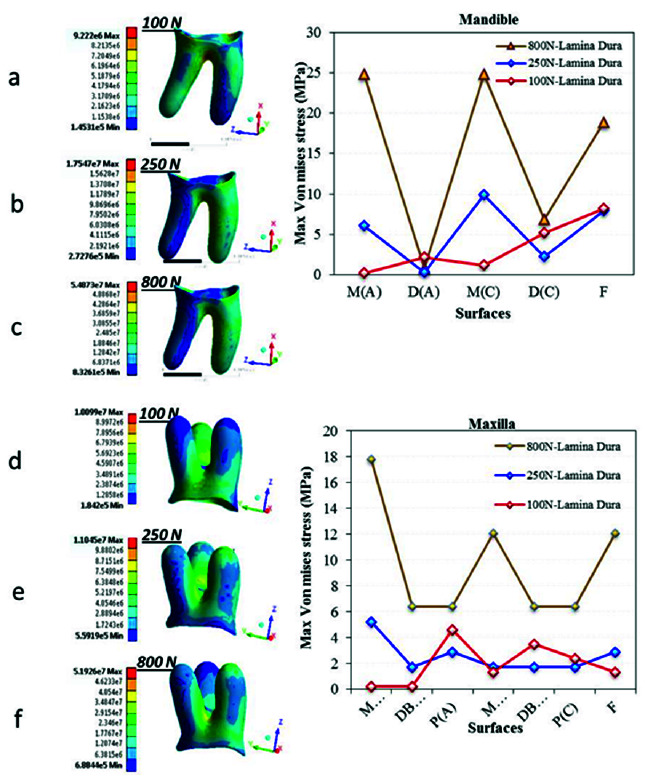
Von Mises stress distribution in the lamina dura for the six models; Figures a, b and c present stress distribution in the mandible under **a:** 100 N oblique load, **b:** 250 N vertical load, and **c:** 800 N traumatic load. Figures d, e and f present stress distribution in the maxilla under **d:** 100 N oblique load, **e:** 250 N vertical load, and **f:** 800 N traumatic load. (Mandible: M(A): Mesial (Apex), D(B): Distal (Apex), M(C): Mesial (Cervical), D(C): Distal (Cervical); F: Furcation). (Maxilla: MB(A)L Mesiobuccal Apex), DB(A): Distobuccal (Apex), P(A): Palatal (Apex), MB(C): Mesiobuccal (Cervical), DB(C): Distobuccal (Cervical), P(C): Palatal (Cervical), F: Furcation)

After passing through the tooth structure, stress reaches the PDL. Figure 5 shows the stress distribution in the PDL. As shown, the magnitude of stress in the PDL was lower than the corresponding value in dental hard tissue, and stress concentration occurred mainly at the interface of cervical third and middle third. The maximum stress concentration was noted in the distal root in model 1, mesial root in models 2 and 3, distal and palatal roots in model 4, mesiobuccal root in model 5, and mesial root in model 6 (Figure 5). The maximum stress in oblique loading of the mandibular first molar was twice the value in the maxillary first molar (Figures 5a and d). In vertical loading, however, the value in the mandibular first molar was higher than that in the maxillary first molar (Figures 5b, c, e, and f). Lamina dura, which is the first layer of bone around the root, receives the distributed stresses prior to complete disintegration of load, as shown in Figure 6. The MVMS value in oblique loading was noted in the distal root and furcation area of the mandibular first molar and palatal root and furcation area of the maxillary first molar. However, in standard vertical and MMF loadings, the MVMS values were concentrated in the middle third of the mesial root of the mandibular first molar, and middle third of the palatal root of the maxillary first molar, as well as the furcation area of both maxillary and mandibular first molars. 

**Figure 7:**
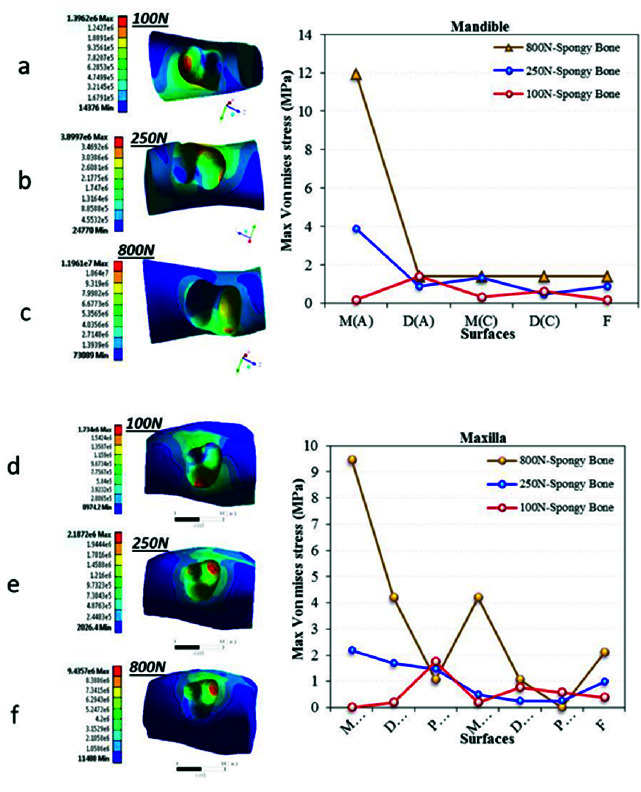
Von Mises stress distribution in the cancellous bone for the six models. Figures a, b and c present stress distribution in the mandible under **a:** 100 N oblique load, **b:** 250 N vertical load and **c:** 800 N traumatic load. Figures d, e and f present stress distribution in the maxilla under **d:** 100 N oblique load, **e:** 250 N vertical load and **f:** 800 N traumatic load. (Mandible: M(A): Mesial (Apex), D(B): Distal (Apex), M(C): Mesial (Cervical), D(C): Distal (Cervical), F: Furcation). (Maxilla: MB(A): Mesiobuccal Apex), DB(A): Distobuccal (Apex), P(A): Palatal (Apex), MB(C): Mesiobuccal (Cervical), DB(C): Distobuccal (Cervical), P(C): Palatal (Cervical), F:Furcation)

The final step of stress disintegration occurred in the cancellous bone (Figure 7). The MVMS value under standard vertical and MMF loadings was concentrated in the apical third around the mesial root of the mandibular first molar and mesiobuccal root of the maxillary first molar. Its magnitude was slightly higher in the maxillary first molar (approximately 2 MPa). However, in oblique loading, stress, in an amount of 2 MPa was noted around the apex of distal root in model 1 and apex of palatal root in model 4. The difference in stress values under vertical and oblique loadings also decreased in the cancellous bone.

The maximum stress in the enamel under standard vertical loading was 162 MPa in the maxillary first molar (three-rooted first molar) and 51 MPa in the mandibular first molar (two-rooted first molar). These values were 2.1 MPa and 3.8 MPa in the cancellous bone in the maxilla and mandible, respectively. Also, the maximum stress in oblique loading decreased from the occlusal surface towards the cancellous bone from 320 MPa to 1.7 MPa in the maxilla, indicating a reduction by 188 times. In the mandible, the maximum stress decreased from 410 MPa in the occlusal surface to 1.3 MPa in the cancellous bone, indicating a reduction by 315 times. 

**Figure 8:**
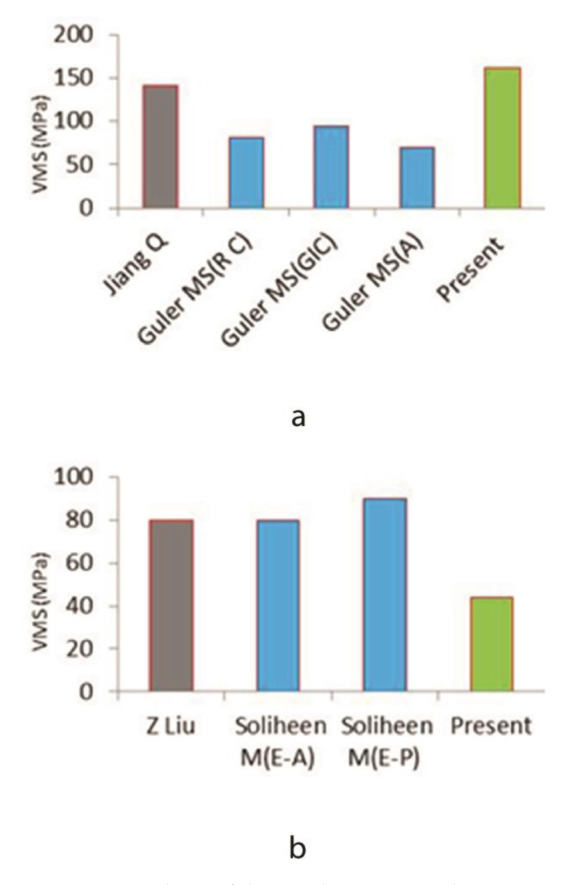
Comparison of the maximum von Mises stress values (MVMS) obtained in the present study with the findings of previous studies, **a:** Maxillary first molar under standard loading, **b:** Mandibular first molar under standard loading. (RC: Resin composite, GIC: Glass ionomer cement, A: Amalgam, E-A: Enamel-Amalgam, E-P: Enamel-Porcelain)

Figure 8a and b shows the MVMS values in maxillary and mandibular first molars under standard loading conditions in the present study in comparison with previous studies on this topic. In this study, the maximum stress under standard loading conditions was 160 MPa in the maxillary first molar and 44MPa in the mandibular first molar. The stress value in the maxillary first molar enamel was approximately 4 times the value in the mandibular first molar enamel. The stress values were approximately tripled under MMF loading (500 MPa in the maxillary first molar and 140 MPa in the mandibular first molar). However, under oblique loading conditions, the concentrated stress in the occlusal surface of the mandibular first molar was higher than that in the maxillary first molar (420 MPa versus 320 MPa). In the study by Jiang *et al*. [ 37 ], the maximum stress value at 5 points in an intact maxillary first molar under 800 N load was approximately 340 MPa, while the MVMS value was 90 MPa in the occlusal surface under oblique loading. They showed higher stress concentration under vertical MMF loading compared with oblique loading. Also, Guler *et al*. [ 26 ] reported that the stress value applied to the occlusal surface of a maxillary first molar with an intact occlusal surface and a class V restoration was 70-90 MPa under masticatory forces (Figure 8a). Similarly, in the mandible, comparisons with some other studies indicated 80 MPa maximum stress accumulation in the occlusal surface of restored teeth [ 49 - 50 ] (Figure 8b).

## Discussion

Evidence shows that the periodontium transfers the masticatory forces to the jawbone during the masticatory function [ 2 ]. However, all teeth and periodontal components need to be separately analyzed to assess the path of disintegration of force and find the susceptible high-stress areas for prosthetic adjustment of multi-rooted teeth. The pattern of stress distribution appears to be different in three-rooted maxillary first molars and two-rooted mandibular first molars. Thus, stress distribution in different parts of maxillary and mandibular first molars and their periodontium was evaluated in the present study. Generally, the masticatory force transfer depends on the tooth support mechanism, which is impaired when the periodontium is damaged [ 51 ]. Thus, periodontal adaptation is affected by the magnitude, direction, duration, and frequency of load application. When the applied occlusal forces exceed the maximum adaptation capacity of a tooth, tissue injury or trauma 

from occlusion may occur [ 52 ]. 

Animal studies have revealed the histological events responsible for this adaptation, which include bone formation at the tension sites and bone resorption at the pressure sites in the periodontium [ 53 - 57 ].Thus, detection of high-stress and tension points and their generalization to the clinical setting seem imperative. In the present study, both occlusal tables received a vertical occlusal force with the same magnitude at 5 points to simulate physiological and MMF loading conditions [ 37 , 45 , 47 - 48 ] and at 2 points for simulation of lateral forces applied to the teeth in function according to previous studies [ 37 , 58 ].

The results of the present study indicated MVMS concentration in the occlusal enamel and cervical dentin (Figures 3 and 4). These areas are in conformity with those reported by the previous studies in different teeth [ 42 - 43 , 59 - 60 ]. The points of MVMS concentration in the enamel are depicted in graphs in Figures 3-8. For a more accurate analysis, the results were compared with the findings of Jiang *et al*. [ 37 ] and Guler *et al*. [ 26 ]. Regarding the MVMS values, the findings of Jiang *et al*. [ 37 ] and Guler *et al*. [ 26 ] were almost consistent with the present results. However, they did not make a comparison between the maxillary and mandibular molar teeth. 

High stress concentration in the enamel, as reported in many studies, is due to the hardness and high modulus of elasticity of the enamel as well as its higher resistance against the applied forces. Thus, enamel tolerates high levels of stress and significantly decreases the magnitude of stress, which is higher in three-rooted first molars under vertical loading compared with two-rooted first molars and *vice versa* under oblique loads (Figure 3).

Considering the lower hardness of dentin than enamel, the magnitude of stress decreased in dentin (Figure 4a). Stress concentration in the maxillary first molar dentin was mainly in the palatal and mesiobuccal roots under standard loading. Under parafunctional forces, the concentration of stress was in the mesiobuccal root, while under oblique loading, stress concentrated in the cervical third of the palatal root. These results were in line with the previous findings [ 61 ]. In the mandibular first molar, however, the masticatory loads were mainly distributed through the buccal surface of the mesial root. Under oblique loading, stress was transferred from the buccal surface of the tooth crown and lingual surface of the distal root [ 62 - 63 ]. This finding has been confirmed by the authors who assessed stress distribution in dentin of endodontically-treated teeth using FEA. They added that considering the stress accumulation points, endodontic posts should be preferably placed in the distal root [ 36 , 45 ]. These changes are due to the fact that the first molar mesial root has a wider buccolingual than mesiodistal dimension, and has an ovoid shape [ 64 ]. Thus, it receives higher stress levels in this dimension, which can lead to vertical fracture in this dimension. On the other hand, considering the morphology and higher concavity of the mesial root than the distal root, and the difference in the load application axis and stress accumulation points between the two roots, higher torque is generated in the mesial root [ 65 - 66 ]. It should be noted that since the root dentin is thin at the apex, cracks often initiate from the apical region in such fractures [ 67 - 68 ]. However, under oblique loading, the maximum stress is accumulated in the cervical third of the distal root, which is probably due to the proximity of dentin to the alveolar bone crest. Bone crest serves as a lever and generates torque, which results in further damage under masticatory loads, causing mainly horizontal fractures [ 63 , 69 ].

In general, the results revealed that the stress concentration point in load transfer from the tooth to the attachment apparatus changes from the cervical dentin towards the middle third in the lamina dura, and then to the apical third in the cancellous bone in two-rooted and three-rooted first molars. In the PDL, stress concentration occurs at the interface of the cervical third and middle third (Figures 5-7). Similarly, Poiate *et al*. [ 69 ] reported stress concentration in the same points in the PDL of a maxillary central incisor. In addition, some researchers reported that the point of maximum stress concentration changed from the coronal third in dentin of a sound tooth to middle or apical third in an endodontically treated tooth [ 45 , 70 - 72 ]. These areas are the sites of maximum collagen fiber degradation and alveolar bone loss. It appears that the oblique fibers in the PDL, mainly located in the middle third of the root, are responsible for this process [ 71 ]. Thus, loads are transferred from the coronal third to the middle third in the lamina dura. Moreover, by the root-end compression in bone, the maximum stress is concentrated around the apex in cancellous bone. On the other hand, stress concentration points may vary in different parts of the tooth and periodontium in the maxilla and mandible, due to variations in number (two or three), location, and morphology of the roots. Therefore, further investigations are warranted on this topic. 

Furthermore, previous studies [ 37 , 39 - 40 ] that evaluated stress distribution in teeth with different access cavity designs and root morphologies reported that the risk of root resorption varied in roots with abnormal and deviated morphologies. This can cause changes in stress distribution. However, in the present study, normal root and crown morphology was considered for evaluation of stress distribution under vertical and oblique loadings. 

The present study had some limitations. For instance, cyclic loading was not simulated because precise simulation of the actual condition and properties of the jawbone is complicated. Stress distribution pattern in bone defect of maxillary and mandibular molars should be investigated in future studies. In addition, not all materials, including the cortical bone and PDL, exhibit completely linear and homogeneous behavior. However, the authors had to consider all materials and models to be linear, homogeneous, elastic, and isotropic. 

### Clinical relevance

Knowledge about the areas that are more susceptible to stress in the posterior teeth can reveal areas susceptible to root fracture under parafunctional forces, and highlights the significance of precise occlusal adjustment following posterior tooth restorations.

## Conclusion

The obtained results highlighted the difference in response of maxillary and mandibular first molars with various root locations under different loading conditions (normal mastication and MMF), indicating a well-organized harmony between the loads applied to the teeth, and their biological response according to their root location. 

## Conflict of Interest

The authors have no conflict of interests regarding this manuscript. 
